# Suitable illumination intensity is essential for preserving the quality of cucumber (*Cucumis sativus* L.) seedlings during storage

**DOI:** 10.1371/journal.pone.0247882

**Published:** 2021-03-05

**Authors:** Juanqi Li, Yang Li

**Affiliations:** College of Horticulture, Henan Agricultural University, Zhengzhou, China; Huazhong Agriculture University, CHINA

## Abstract

Continuous darkness decreases seedling quality during storage, whereas appropriate light quality and intensity can overcome these negative effects. In this study, we determined the light intensity, storage time (ST), and storage temperature suitable for cucumber (*Cucumis sativa* L.) seedlings. We stored cucumber seedlings under four different photosynthetic photon flux densities (PPFDs; 0, 15, 30, and 45 μmol·m^-2^·s^-1^) at 12°C, and examined how the morphological, physiological, and photosynthetic changes in seedlings during storage affected their ability to recover after transplanting. Our results indicated that at least 15 μmol·m^-2^·s^-1^ PPFD was needed for cucumber seedlings stored in the dark for 2 or 4 d, and at least 30 μmol·m^-2^·s^-1^ PPFD was needed when the ST was extended to 6 d. Overall, our results showed that cucumber seedlings require light-emitting diode (LED) illumination during storage to maintain their quality and recovery ability.

## 1. Introduction

Cucumber (*Cucumis sativus* L.; Cucurbitaceae) is an important crop worldwide. China is the largest producer of cucumber, accounting for approximately 77% of global cucumber production [1]. With the industrialization of vegetable production, the demand for cucumber seedlings has increased. The process of seedling production is affected by issues such as sales, weather, and human factors, and therefore the phenomenon by which seedlings reach their transplanting age or commercialization standard but cannot be transplanted on time occurs frequently [2–4]. The duration of storage directly affects the quality of seedlings and consequently their growth after transplantation. Therefore, maintaining a high quality during storage and after transplantation is critical.

Storage of seedlings is a relatively stressful process, as their “source-library” balance is destroyed, resulting in a variety of morphological, physiological, and metabolic changes, which degrade seedling quality. In China, most seedlings are stored in dark conditions at low temperature; however, continuous darkness decreases seedling quality and causes leaf yellowing [5, 6], leaf abscission [7], plasma membrane peroxidation [7, 8], and carbohydrate reduction [2], which affects growth after transplantation.

A combination of low temperature and dim light has been shown to maintain seedling quality [9]. Kubota and Kozai reported that the most suitable storage conditions for seedlings was the absence of any CO_2_ exchange during the whole storage period and showed that over 24 h of continuous light, the light compensation point was the most suitable light intensity [9]. Furthermore, maintaining a constant photosynthetic photon flux density (PPFD) helps to maintain the dry weight of seedlings and suppresses the decline in seedling quality [5]. A previous study showed that the light compensation point of cucumber seedlings is approximately 7.5 μmol·m^-2^·s^-1^ at 10°C and approximately 8.3 μmol·m^-2^·s^-1^ at 13°C [4]. Therefore, we speculated that the light compensation point of cucumber seedlings at 12°C is approximately 8 μmol·m^-2^·s^-1^. It is important to preserve seedling quality during storage, i.e., inhibiting succulent elongation, leaf yellowing, and wilting, as well as the regrowth ability, photosynthetic performance [4, 7], and the relative growth rate (RGR) of new leaves [7] after storage.

In China, there have been few studies of light use during seedling storage, although this will affect the photosynthetic apparatus of the plants [4, 10]. Several reports have investigated seedling deterioration during storage [7, 10–12], but only a few studies have been conducted on the restoration of growth parameters such as morphology and photosynthesis. Furthermore, to the best of our knowledge, all previous studies have used lamps as the light source. In comparison, light-emitting diodes (LEDs) offer many advantages over lamps because of their high efficiency, lower calorie requirement, compact size, and good spectral performance [13]. However, the optimum LED illumination conditions for the preservation of the quality of cucumber plug seedlings during storage have not yet been investigated in cucumber plug seedlings.

The aim of this study was to determine the most suitable light intensity and storage time (ST) at an appropriate storage temperature for cucumber seedlings. Moreover, we investigated how the morphological, physiological, and photosynthetic changes in seedlings during storage affected their recovery after transplantation.

## 2. Materials and methods

### 2.1 Plant materials and growth conditions

Seeds of the cucumber cultivar ‘Zhong Nong 16’ were sown in fifteen 72-cell plastic plug trays (50 cm^3^) filled with a substrate comprising peat, vermiculite, and perlite in the ratio of 2:1:1 (v/v/v). The trays were placed in a greenhouse under a natural photoperiod, with day/night temperatures of 25 ± 3/16 ± 3°C. Uniform seedlings with one fully expanded true leaf and one small true leaf were selected 25 days after sowing and transferred to an artificial climate chamber maintained at 25 ± 2/18 ± 2°C day/night temperature, 60 ± 10% relative humidity, 12-h light/12-h dark photoperiod, and 250 ± 30 μmol·m^-2^·s^-1^ PPFD. Subirrigation was applied with full-strength Yamazaki nutrient solution [14].

### 2.2 Storage and transplant conditions

After one week of growth in the artificial climate chamber (Grandcool, Beijing, China), 12 trays were moved to another artificial climate chamber, and seedlings were grown under three different PPFDs (0, 15, 30, and 45 μmol·m^-2^·s^-1^) and a 12-h photoperiod, while the remaining three trays were left behind as controls (250 μmol·m^-2^·s^-1^ PPFD). Light quality is a combination of red and blue light (red/blue light PPFD ratio is 8), in which the red monochromatic light with a maximum intensity at 660 nm and blue monochromatic light with a maximum intensity at 440 nm. Red and blue lights consists of a panel and a control system (Red: YYC-FA01REM140; Blue: YYC-FA01BLM140, YYC Optoelectronics, China).Light was provided during storage using LEDs, which were installed 30 cm above the seedling canopy. The air temperature was set to 12 ± 1°C. Three trays were used for each PPFD treatment. Each day, subirrigation was applied with a full-strength Yamazaki nutrient solution for 5 min under dim light (<1 μmol·m^-2^·s^-1^) at the artificial climate chamber temperature.

At 0, 2, 4, and 6 d of storage, 18 seedlings from three trays in each treatment were transplanted into 10-cm diameter plastic pots and placed in the original artificial climate chamber, and recovery of the seedlings was monitored for 8 d. Seedlings were supplied with full-strength Yamazaki nutrient solution once per day.

### 2.3 Measurements

Seedlings were sampled at 0, 2, 4, and 6 d to measure various indexes, including stem length, stem diameter, fresh and dry shoot and root weights, leaf health index, leaf chlorophyll concentration, soluble sugar content, chloroplast ultrastructure, and net photosynthesis rate (Pn). After 8 d of recovery, the plant height, stem diameter, number of leaves, fresh and dry weights of shoots and roots, RGR of new leaves, and Pn of the transplanted seedlings were measured.

#### 2.3.1 Shoot length and stem diameter

Seedling length was measured from the base of the cotyledon to-apical point along a straight line. Shoot diameter (longer side) was measured at 1 cm below the cotyledon using an electronic Vernier caliper. Nine plants from each treatment were used for these measurements.

#### 2.3.2 Leaf health index

Leaf health index values were calculated using 18 plants per treatment during storage, and leaf blade degeneration could be divided into several levels (Table 1) [7].

**Table 1:** Levels of leaf degeneration in cucumber seedlings.

Level	Cotyledon phenotype	True leaf phenotype
0	Normal development, no yellowing or wilting	Normal development
1	Normal development, partial yellowing (10%), not deciduous	Normal development
2	Slight yellowing (10%), partial wilting (10%)	Normal development
3	Whole cotyledon yellow, largely deciduous (70%)	Normal development
4	Almost all cotyledon deciduous (> 90%)	Wilting or yellowing of first and second true leaves
5	Completely deciduous	Yellowing or wilting of most true leaves

The leaf degradation index (LDI) of cucumber seedlings was calculated using the following equation:

$$LDI=\frac{\sum xa}{n\sum x}=\frac{\sum x_{1}a_{1}+x_{2}a_{2}+x_{3}a_{3}+\ldots +x_{n}a_{n}}{n\sum x}$$

where x_1_, x_2_, x_3_, and x_n_ represent the number of cucumber seedlings at each level; and a_1_, a_2_, a_3_, and a_n_ represent the different levels.

The leaf integrity index (LII) of cucumber seedlings was estimated as follows:

LII=1−LDI

#### 2.3.3 Chlorophyll concentration

Leaf disks (0.84 cm diameter) were collected from six plants, and eight disks per plant were weighed and used for the measurements. Chlorophyll was extracted from the cotyledon and first true leaf using 95% ethyl alcohol in the dark for 24 h. The chlorophyll content was determined by measuring the absorbance at 665 and 649 nm using an ultraviolet-visible (UV-Vis) spectrophotometer, as described previously [15]. Subsequently, the eight disks from each plant were placed in folded paper envelopes and dried at 85°C for 48 h to determine their dry weight.

#### 2.3.4 Soluble sugar content

The first leaves were frozen in liquid N_2_ and then thawed for a soluble sugar content analysis, as described previously [16], using a UV-Vis spectrophotometer.

#### 2.3.5 Electron microscopy

The central portion of the leaf blade, without veins, was cut into rectangles (approximately 2 × 1 mm) and used to observe the chloroplast ultrastructure as described previously [17].

#### 2.3.6 Photosynthetic gas exchange

The Pn, stomatal conductance (Gs), intercellular CO_2_ concentration (Ci), and transpiration rate (Tr) were measured using the LI-6400xt system (Li-COR Inc., Lincoln, NE, USA) equipped with an LED light source at 250 μmol·m^-2^·s^-1^ PPFD. The leaf area clipped by the chamber was 6 cm^2^. The temperature, relative humidity, and CO_2_ concentration were maintained at 25°C, 40 ± 5%, and 400 μmol·mol^-1^, respectively, during the measurements. At least three plants from each treatment were used for these measurements.

#### 2.3.7 The RGR of new leaves after transplanting

After transplanting, 18 plants from each treatment were numbered. The location of the heart of each plant was marked and outlined using a transparent paper to obtain the heart leaf area (S1 Data). After 8 d of recovery, the new leaves of each plant were cut using an Epson Expression 4990 root system scanner (Epson, Tokyo, Japan) to obtain the leaf area (S1 Data). Then, the RGR of new leaves was calculated as follows [18]:

$$RGR=\frac{\left(lnS2-lnS1\right)}{8}$$

## 3. Results

### 3.1 Morphological changes in cucumber seedlings during storage

**Fig 1:**
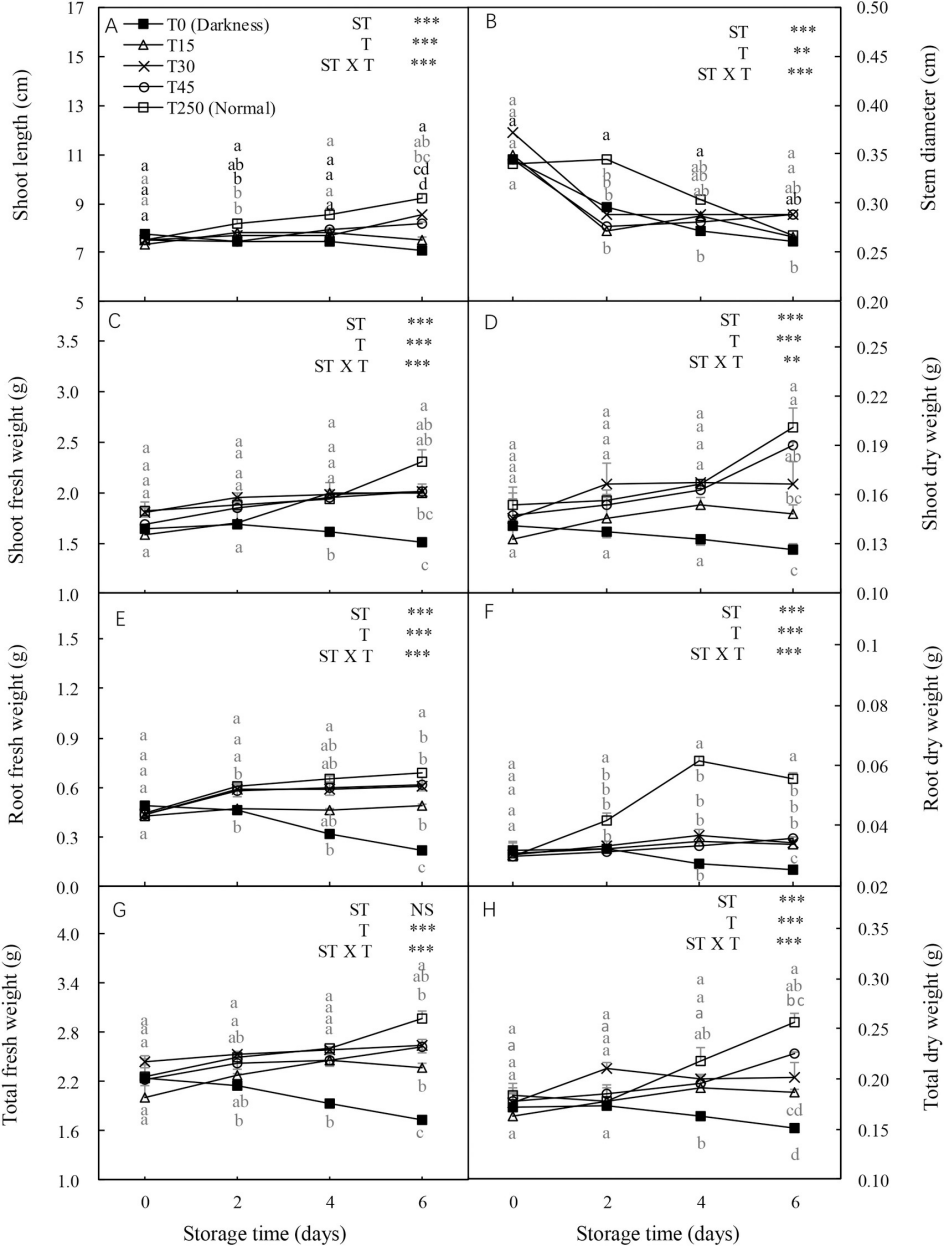
Effect of storage duration on morphological changes in cucumber seedlings. Seedlings were stored at 12°C for 0, 2, 4, or 6 d in the dark (T0) or under a 15, 30, and 45 μmol·m^-2^·s^-1^ photosynthetic photon flux density (PPFD; T15, T30, and T45, respectively). Seedlings labeled as normal were grown under 250 μmol·m^-2^·s^-1^ PPFD (T250). Data represent the mean ± standard error (SE) of nine replicates. Different lowercase letters indicate significant differences among treatments on a given day according to the Tukey test (*P* < 0.05). ST, storage time; T, treatment.

The shoot length, stem diameter, shoot and root fresh weights, and shoot, root, and total dry weights were significantly influenced by the ST and light intensity treatment (T) and their interaction. In the dark (T0), most seedling growth parameters, including stem diameter, shoot fresh and dry weights, and total fresh and dry weights, showed decreasing trends with increased ST (Fig 1). However, the adverse effects of darkness on seedlings during storage were alleviated by illumination. After 4 d of storage, most parameters, including shoot fresh and dry weights and total fresh and dry weights, were increased by the light treatment compared with the T0 seedlings. Similar trends were observed after 6 d of storage. Additionally, no significant differences were detected in most parameters between T15 and T250 (normal culture conditions) seedlings after 2 and 4 d of storage, or between T45 and T250 seedlings after 6 d of storage.

### 3.2 Chlorophyll concentration and leaf health index of seedlings during storage

**Fig 2:**
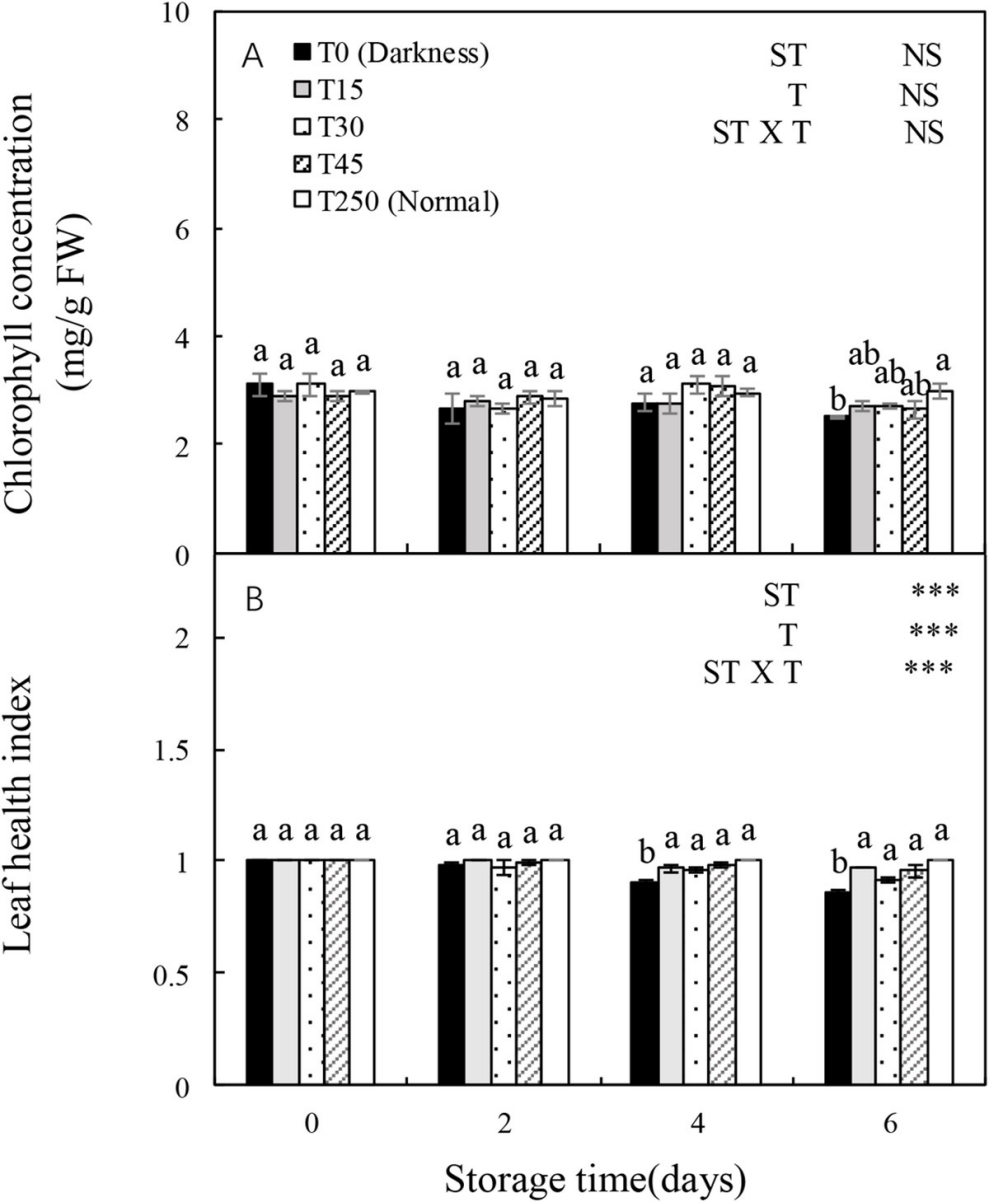
Changes in the chlorophyll concentration and leaf health index of cucumber seedlings during storage under different light conditions at 12°C. Control seedlings were grown under normal conditions (T250). Data represent the mean ± SE (*n* = 9). Different lowercase letters indicate significant differences among treatments on a given day according to the Tukey test (NS, non-significant; ***, *P* < 0.001; **, *P* < 0.01). ST, storage time; T, treatment.

The leaf health index, but not the chlorophyll content, was significantly influenced by ST, T, and the ST × T interaction (Fig 2). The chlorophyll concentration was relatively stable after 4 d of storage; however, after 6 d of storage, it decreased significantly in the T0 treatment but not in the T15, T30, and T45 treatments in comparison with T250 seedlings (Fig 2A). Moreover, the leaf health index showed a decreasing trend with increased ST in the dark (T0). After 4 d of storage, the T15, T30, and T45 treatments produced a significantly higher leaf health index compared with T0, but not when compared with T250 seedlings. The leaf health index after 6 d of storage showed similar trends with the trends after 4 d of storage.

### 3.3 Ultrastructure of chloroplasts in cucumber leaves during storage

**Fig 3:**
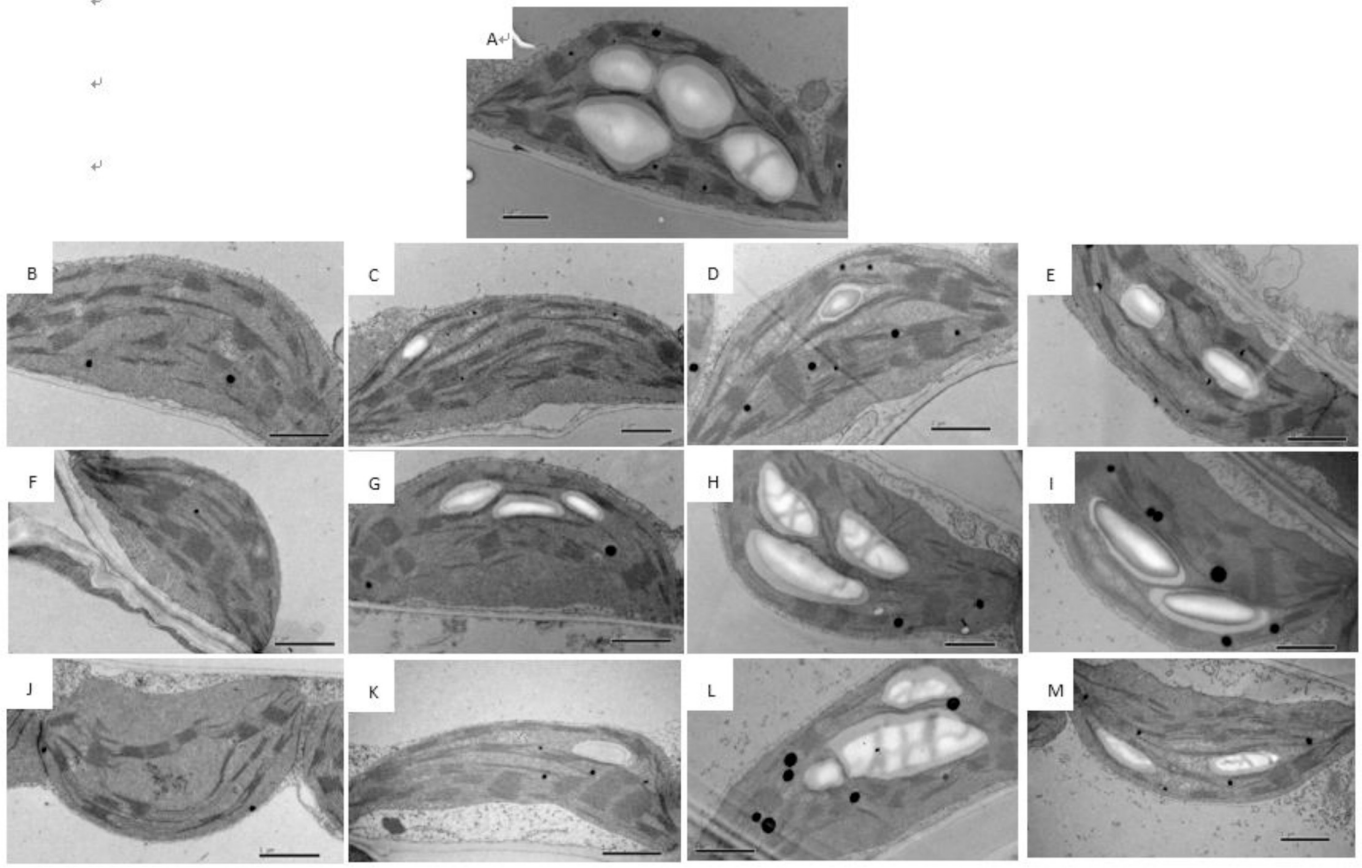
Changes in chloroplast ultrastructure in the leaves of cucumber seedlings stored under different light conditions at 12°C. (A–M) Seedlings were stored for 0 d (A), 2 d (B–E), 4 d (F–I), and 6 d (J–M) in the dark (B, F, J) or under 15 μmol·m^-2^·s^-1^ PPFD (C, G, K), 30 μmol·m^-2^·s^-1^ PPFD (D, H, L), or 45 μmol·m^-2^·s^-1^ PPFD (E, I, M) at 12°C. Scale bar: 1 μm. SG, starch grain; G, grana thylakoid.

The ultrastructure of cucumber leaf chloroplasts was significantly influenced by ST and T. Before storage (0 d), leaf chloroplasts were elliptical in shape and were filled with starch grains, and the chloroplast grana thylakoids were closely arranged (Fig 3A). In contrast, the chloroplasts of dark-stored leaves were round in shape and lacked starch grains, and the grana thylakoids decreased in number and were ambiguous (Fig 3B, 3F and 3J). However, the ultrastructure of chloroplasts in cucumber leaves was maintained when stored under light, when compared with T0 seedlings. Starch grains in the chloroplasts of leaves under light were fewer in number and smaller compared with non-stored seedlings. No significant differences were detected in the chloroplasts between three light-stored and non-stored seedlings within 4 d of storage (Fig 3).

### 3.4 Soluble sugar content of seedlings during storage

The soluble sugar content of cucumber seedlings was significantly affected by ST, T, and the ST × T interaction. The soluble sugar content of seedlings in the T0 and T15 treatments decreased with increasing ST. However, providing seedlings with appropriate light during storage alleviated the adverse effects. After 4 d of storage, T0 seedlings had a significantly lower soluble sugar content than T250 seedlings, whereas there were no significant differences in T15, T30, and T45 seedlings compared with T250 seedlings. After 6 d of storage, seedlings in all treatments had a significantly lower soluble sugar content than T250 seedlings, although T30 and T45 seedlings maintained a higher soluble sugar content than T0 seedlings.

### 3.5 Photosynthetic parameters of cucumber seedlings during storage

**Fig 4:**
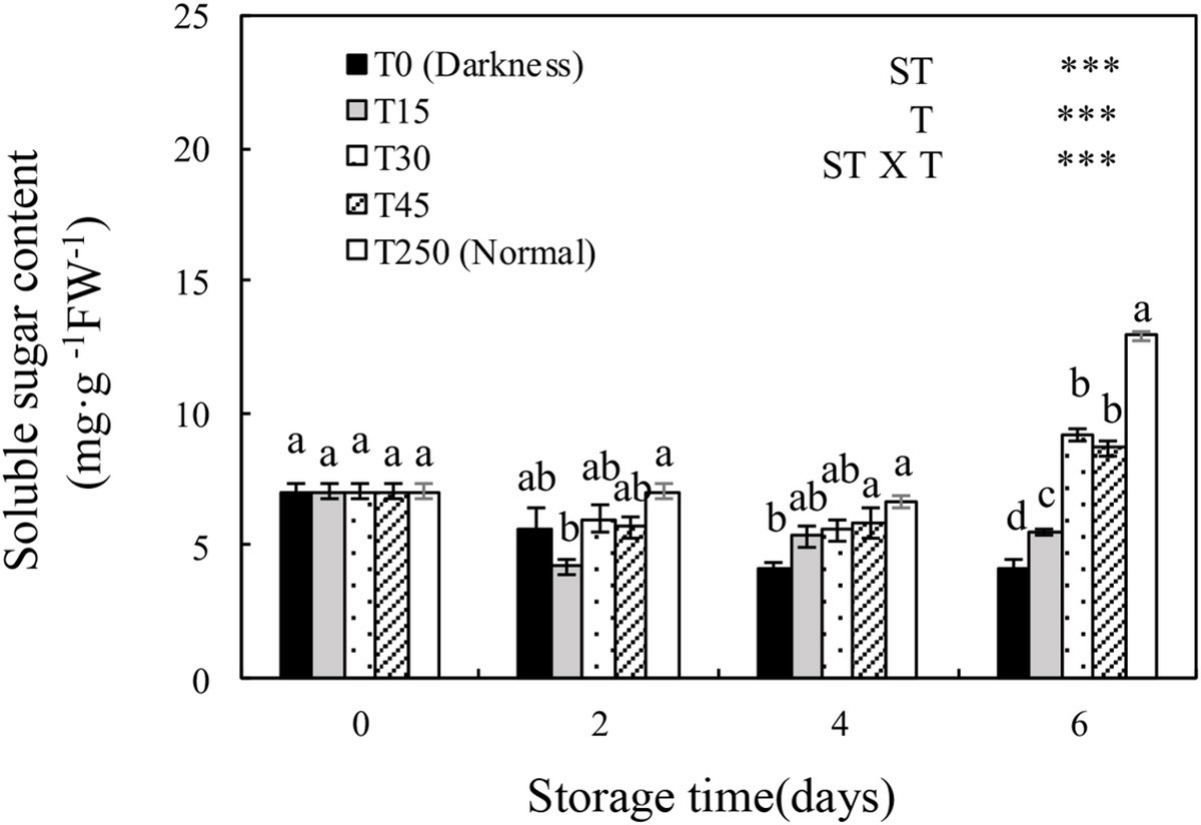
Changes in the soluble sugar content of the leaves of cucumber seedlings during storage under different light conditions at 12°C. Control seedlings were grown under normal conditions (T250). Data represent the mean ± SE (*n* = 9). Different lowercase letters indicate significant differences among treatments on a given day according to the Tukey test (NS, non-significant; ***, *P* < 0.001; **, *P* < 0.01). ST, storage time; T, treatment.

Both Pn and Gs were significantly affected by ST, T, and the ST × T interaction (Fig 4A and 4B). Both Pn and Gs decreased with increasing ST under the dark condition (T0); however, this was alleviated by providing light during storage. After 4 d of storage, T15 seedlings had a significantly higher Pn than T0 seedlings, but there no significant differences compared with T250 seedlings. After 6 d of storage, there were no significant differences in T30 and T45 seedlings compared with T0, T15, and T250 seedlings (Fig 4A). There were similar trends in Gs (Fig 4B). In addition, there were no significant differences in Ci among treatments after 6 d of storage (Fig 4C). There was a decreasing trend in Tr with increased ST in all treatments. After 6 d of storage, there were no significant differences in T30 and T45 seedlings compared with T250 seedlings.

### 3.6 The RGR of new leaves after transplanting

**Fig 5:**
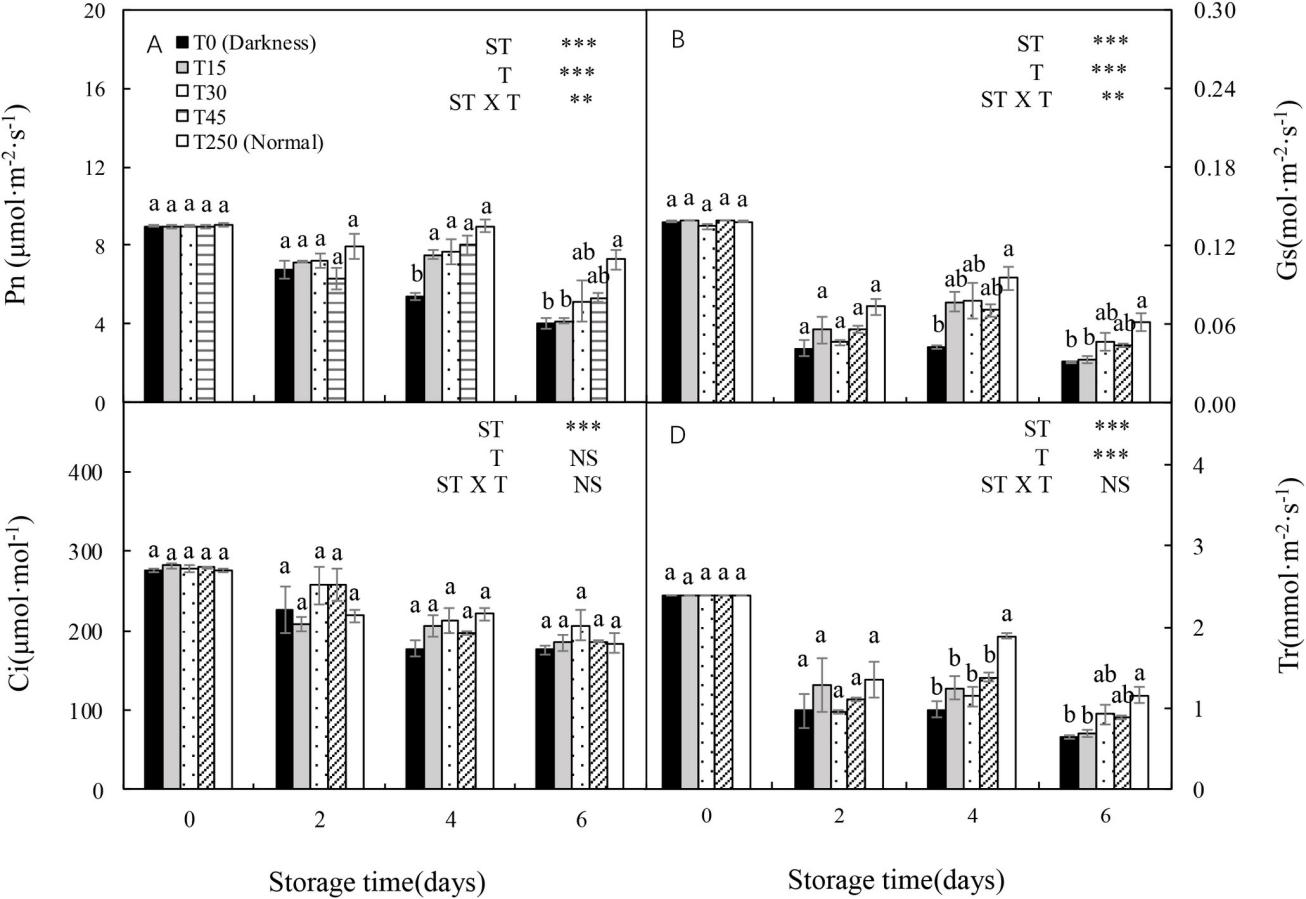
Changes in the net photosynthesis rate (Pn), stomatal conductance (Gs), intercellular CO_2_ concentration (Ci), and transpiration rate (Tr) of cucumber leaves during storage under different light conditions at 12°C. (A–D) Changes in Pn (A), Gs (B), Ci (C), and Tr (D) are shown. Control seedlings were grown under normal conditions (T250). Data represent the mean ± SE (*n* = 9). Different lowercase letters indicate significant differences among treatments on a given day according to the Tukey test (NS, non-significant; ***, *P* < 0.001; **, *P* < 0.01). ST, storage time; T, treatment.

The RGR of new leaves was significantly influenced by ST, T, and their interaction (Fig 5). In the T0 treatment, the RGR of new leaves decreased with an increase in ST. This decrease in RGR was alleviated by providing seedlings with light during storage. There was a significantly higher RGR in new leaves of T15 seedlings than in T0 seedlings after 4 d of storage, but there were no significant differences compared with T250 seedlings. Similar RGR trends were detected in T45 seedlings after 6 d of storage, and there were no significant differences in T30 seedlings compared with T45 seedlings.

### 3.7 Morphological changes in seedlings after transplanting

**Fig 6:**
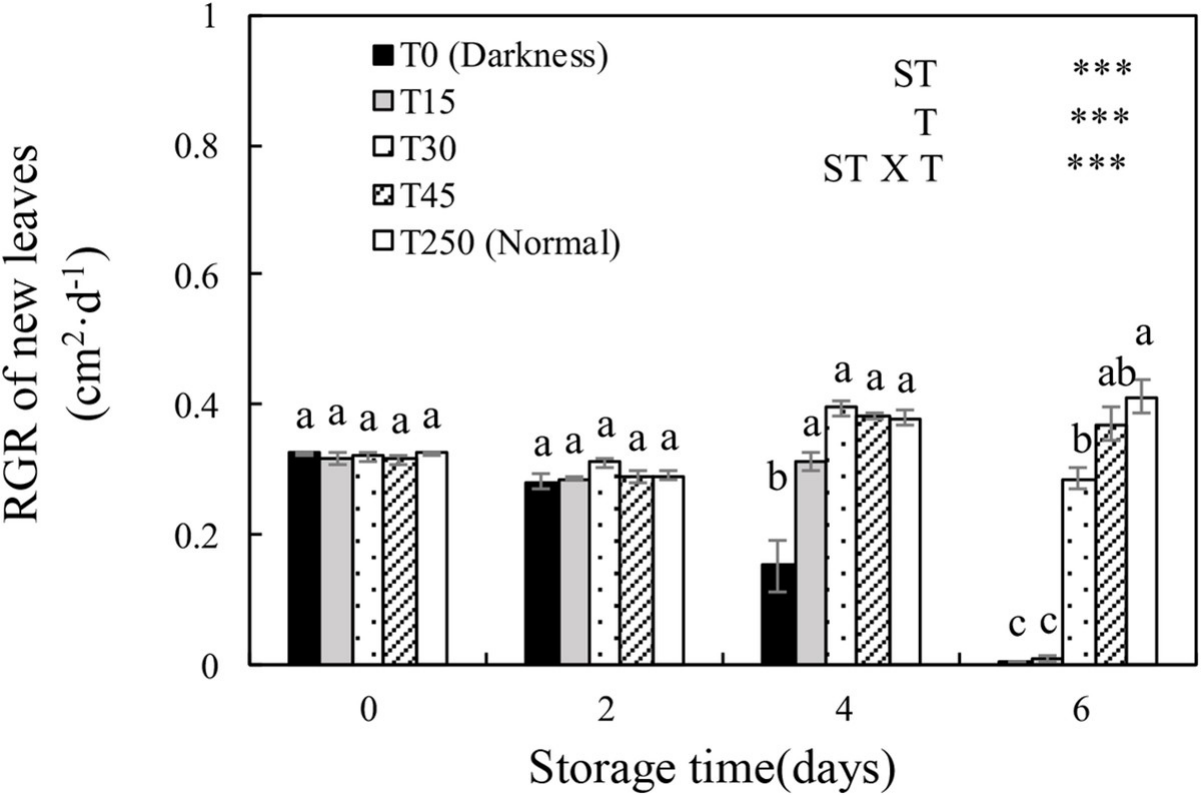
Recovery of the relative growth rate (RGR) of new leaves of cucumber seedlings after storage under different light conditions at 12°C. Data represent the mean ± SE (*n* = 9). Different lowercase letters indicate significant differences among treatments on a given day according to the Tukey test (*, *P* < 0.05; **, *P* < 0.01; ***, *P* < 0.001). ST, storage time; T, treatment.

Shoot length, stem diameter, and shoot, root, and total fresh and dry weights of transplanted seedlings were significantly influenced by ST, T, and their interaction (Fig 6). In the dark (T0), all seedling growth related parameters showed decreasing trends with an increase in ST. However, the adverse effects of darkness were alleviated by illuminating them during storage. After 4 d of storage, most parameters, including shoot length, and shoot, root, and total dry weights, were higher in light treated seedlings than in dark-grown (T0) seedlings. Similar trends were observed after 6 d of storage. Additionally, for most parameters there were no significant differences between T15 and T250 seedlings after 2 and 4 d of storage.

### 3.8 Photosynthetic parameters of seedlings after transplanting

**Fig 7:**
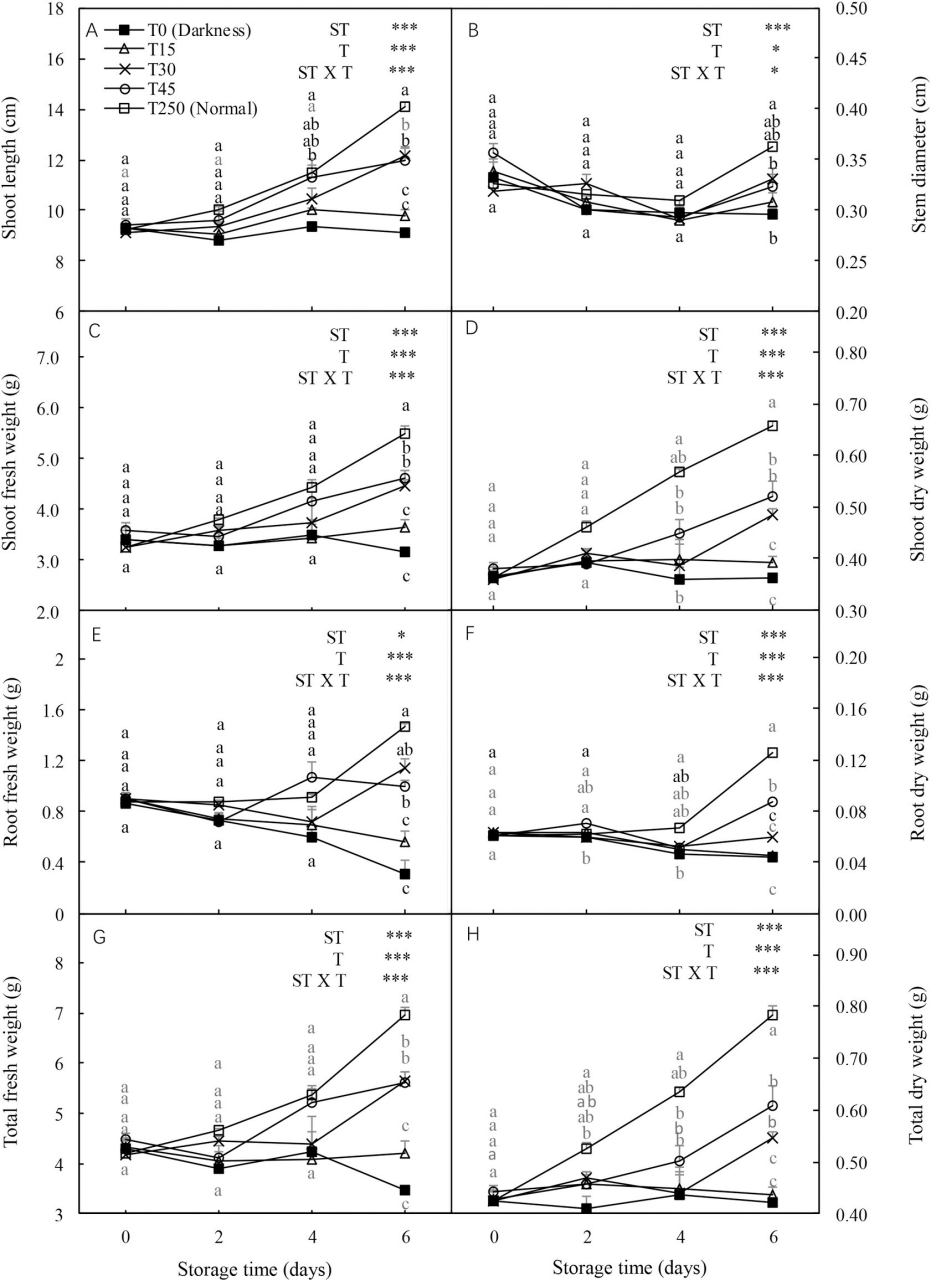
Effect of storage duration on morphological changes in cucumber seedlings after transplanting. Seedlings were stored at 12°C under different light intensities (T0, T15, T30, and T45) for 0, 2, 4, and 6 d. Seedlings labeled as normal were grown under 250 μmol·m^-2^·s^-1^ PPFD (T250). Data represent the mean ± SE (*n* = 9). Different lowercase letters indicate significant differences among treatments on a given day according to the Tukey test (*P* < 0.05). ST, storage time; T, treatment.

Both the Pn and Gs of transplanted seedlings were significantly affected by ST, T, and their interaction (Fig 7A and 7B). The Pn and Gs of T0 seedlings decreased following an increase in ST. However, these adverse effects were alleviated by providing illumination to seedlings during storage. After 4 d of storage, T15 seedlings had a significantly higher Pn than T0 seedlings, but there were no significant differences in Pn compared with T250 seedlings. Similar Pn trends were observed in T30 and T45 seedlings after 6 d of storage. In addition, T30 and T45 seedlings had a higher Gs than T0 and T15 seedlings after 6 d of storage, but there were no significant differences compared with T250 seedlings. Furthermore, there were no significant differences in the Ci and Tr of the transplanted seedlings compared with the T250 seedlings after 6 d of storage.

## 4. Discussion

### 4.1 LED illumination preserves the morphology and leaf health of cucumber seedlings during storage

Low temperature and darkness are commonly used to maintain seedling quality during short-term storage [19]. However, when seedlings are stored in the cold and darkness, photosynthesis ceases, but respiration continues; thus decreasing the dry weight and chlorophyll content of the seedlings [20]. In this study, chlorophyll concentration, and the shoot, root, and total dry weights of cucumber seedlings decreased in the dark (Figs 1 and 2), which was consistent with the results of previous studies on watermelon [4] and tomatoes [7] plug seedlings. This probably occurred because the ultrastructure of leaf chloroplasts changed during dark storage, resulting in the degradation of chlorophyll. Notably, after 4 d of storage, the leaf health index of dark-stored cucumber seedlings decreased significantly, whereas the chlorophyll concentration did not. This was consistent with the observation that the cotyledon started turning yellow after 4 d of dark storage, whereas the first true leaf turned yellow after 6 d of dark storage. The presence of reactive oxygen species (ROS) is a major factor that accelerates chlorophyll degradation. The influence of ROS is directly reflected by changes in membrane integrity [21]. In this study, the shoot, root, and total dry weights of light-stored seedlings were well maintained throughout the experiment, with a slight increase compared with T0 seedlings, but this did not cause the shoot length to increase rapidly (Fig 1). Furthermore, no significant differences were detected in the chlorophyll concentration and leaf health index between light-stored seedlings (T15, T30, and T45) and normally grown seedlings (T250) (Fig 2). These results indicate that at least 15 μmol·m^-2^·s^-1^ PPFD is necessary for maintaining seedling dry weight and leaf health.

### 4.2 LED illumination preserves leaf chloroplast ultrastructure and photosynthetic performance in cucumber seedlings during storage

Light affects chloroplast development, chloroplast structure, and consequently photosynthesis [22–24]. In this study, the chloroplasts in dark-stored seedlings gradually assumed a round shape and lost their starch grains, and the grana thylakoids decreased in number and became ambiguous during storage (Fig 3B, 3F and 3J). Similar results have been previously reported in watermelon seedlings [4]. In cucumber seedlings stored under light (T15, T30, and T45) for 4 d, the chloroplasts assumed a relatively normal shape compared with non-stored seedlings (T250) (Fig 3C–3E and 3G–3I). However, after 6 d of storage, chloroplasts became narrow in T15 seedlings but maintained their shape in T30 and T45 seedlings. In T30 and T45 seedlings there were no significant differences in chloroplast shape during 4 d of storage (Fig 3K–3M).

With an increase in dark ST, the seedlings showed a decrease in carboxylation efficiency and net photosynthetic rate [7, 25]. In our study, Pn and Gs significantly decreased after 4 d of dark storage compared with normally grown leaves (T250). After 6 d of storage, the Pn and Gs of both T0 and T15 leaves decreased significantly compared with T250 leaves (Fig 4A and 4B), probably because of chlorophyll degradation and the altered chloroplast structure (Figs 2 and 3). Similar results have been reported previously [4, 26]. These results indicate that at least 30 μmol·m^-2^·s^-1^ PPFD is necessary for maintaining photosynthesis during 6 d of storage.

**Fig 8:**
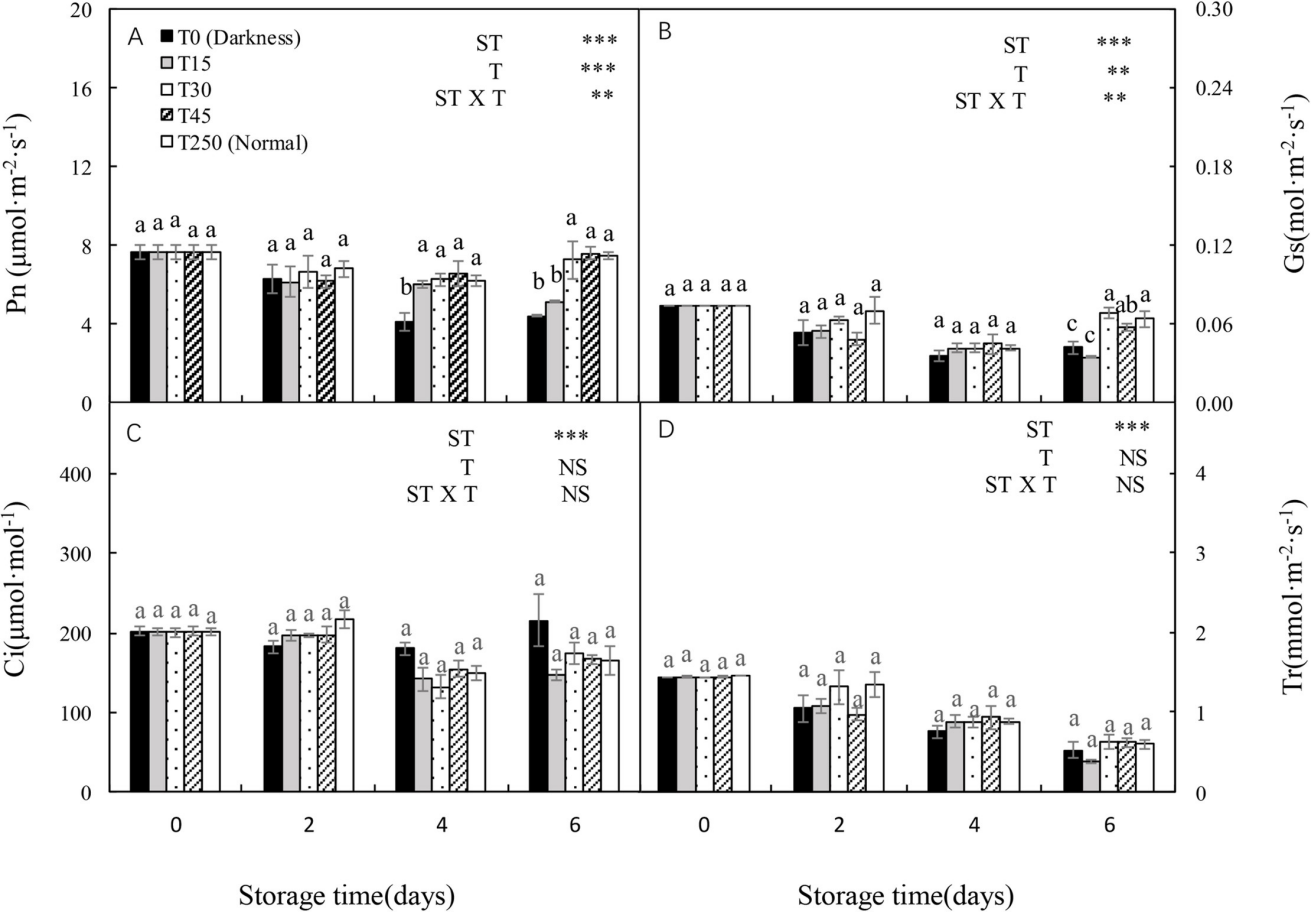
Recovery of photosynthetic parameters of the leaves of cucumber seedlings stored at 12°C under different light intensities. (A) Pn; (B) Gs; (C) Ci; Tr (D). Control seedlings were grown under normal conditions (T250). Data represent the mean ± SE (*n* = 3). Different lowercase letters indicate significant differences among treatments on a given day according to the Tukey test (NS, non-significant; ***, *P* < 0.001; **, *P* < 0.01). ST, storage time; T, treatment.

When seedlings are stored in darkness, photosynthesis ceases and the carbohydrate reserves in plant tissues are remobilized and utilized [27]. In our study, seedlings stored in darkness had a significantly decreased soluble sugar content after 6 d of storage. This reduction in soluble sugar content was alleviated when illuminated by 30 and 45 μmol·m^-2^·s^-1^ PPFD (Fig 8), suggesting that at least 30 μmol·m^-2^·s^-1^ PPFD is necessary for maintaining the soluble sugar content of seedlings during 6 d of storage. In a previous study, cabbage seedlings that accumulated large amounts of soluble sugars during storage showed a higher photosynthetic rate after transplanting [28]. The exogenous application of glucose to watermelon plug seedlings increased carbohydrate accumulation, which increased the performance of seedlings after transplanting [29]. Our experiment also confirmed that the soluble sugar content of seedlings during storage was closely related to the recovery of seedlings after transplanting.

### 4.3 LED illumination preserves the RGR of new leaves and growth of cucumber seedlings after transplanting

The key significance of storage is to ensure that seedlings have a good recovery ability after transplanting, and the RGR of new leaves is an important indicator used to measure plant growth [7, 30]. In this study, after 4 d of storage, the RGR of the new leaves of cucumber seedlings under light could recover to normal culture conditions. When the ST was extended to 6 d, T0 and T15 seedlings almost stopped growing, whereas T30 and T45 seedlings showed normal growth (Fig 5). This was probably because the accumulation of a sufficient amount of carbohydrates during storage enabled the growth and recovery of plants (Figs 1 and 8). These results indicated that at least 30 μmol·m^-2^·s^-1^ PPFD was necessary for maintaining the RGR of new leaves during storage for 6 d.

In early reports, plants grown (fresh and dry weight) under dim light during storage tended to show better growth after transplanting than those grown under darkness [2, 5]. In our study, the shoot length, shoot, root, and total fresh weights, and shoot and total dry weights of T30 seedlings after 6-d storage significantly increased after transplanting compared to those of T0 seedlings (Fig 6). This may be because the shoot length, shoot and root weights (fresh and dry weight), and soluble sugar content of seedlings stored in the dark decreased over time (Figs 1 and 8), which significantly suppressed the growth of seedlings after storage. These results indicate that at least 30 μmol·m^-2^·s^-1^ PPFD during 6-d storage is necessary for maintaining the RGR of new leaves and the growth of plants after transplanting.

### 4.4 LED illumination during storage preserves the photosynthetic performance of cucumber seedlings after transplanting

Exposure of seedlings to light during storage is one of the main factors that preserves their photosynthetic ability; thus, ensuring the transplant quality of post-storage seedlings [3, 28, 31]. In our study, seedlings stored in light conditions had a significantly higher Pn than those stored in darkness after 4 d of storage. However, after 6 d of storage, there were no significant differences in Pn, Gs, Ci, and Tr in T30 and T45 seedlings compared with T250 seedlings (Fig 7), possibly because of higher photosynthesis in T30 and T45 seedlings during storage (Fig 4A). This result demonstrated the preservation of the photosynthetic ability of seedlings stored under an appropriate light intensity, which further ensured the quick recovery of seedlings after transplanting. These results indicate that at least 30 μmol·m^-2^·s^-1^ PPFD is necessary for maintaining photosynthesis after the transplantation of seedlings subjected to 6 d of storage.

## 5. Conclusions

Overall, this study demonstrates that to preserve the quality of cucumber seedlings during storage and to improve their recovery post-transplantation, appropriate LED illumination should be provided when the storage duration changes. Our data showed that cucumber seedlings stored at 12°C in darkness for 2 or 4 d required at least 15 μmol·m^-2^·s^-1^ PPFD, and when the ST was extended to 6 d, at least 30 μmol·m^-2^·s^-1^ PPFD was required to maintain a normal chloroplast ultrastructure, healthy leaves, and optimal photosynthetic performance during storage. The provision of these light intensities would enable cucumber seedlings to recover quickly after transplanting, which would increase the RGR of new leaves, plant growth, and photosynthetic ability.

## Supporting information

S1 Data (XLSX)
